# REDD1 (regulated in development and DNA damage-1)/autophagy inhibition ameliorates fine particulate matter (PM2.5) -induced inflammation and apoptosis in BEAS-2B cells

**DOI:** 10.1080/21655979.2021.1917227

**Published:** 2021-04-29

**Authors:** Yan Li, Xiaoxiao Xu, Liyan Wang, Xuemei Li, Running Liu, Li Zhang, Yali Xu

**Affiliations:** a Department of Pediatric Outpatient, Chongqing Health Center for Women and Children, Chongqing, China; b Department of Pediatric Center, The Third Affiliated Hospital of Chongqing Medical University, Chongqing, China; c Department of Child Health Care, The Fifth People’s Hospital of Chongqing, Chongqing, China

**Keywords:** REDD1, PM2.5, lung injury, autophagy, inflammation

## Abstract

This study aimed to investigate the implication of REDD1 on airborne particle matter-induced lung injury and whether it is mediated through autophagy. Cell viability in BEAS-2B cells induced by PM2.5 was measured by CCK-8. RT-qPCR and Western blot were performed to determine mRNA and protein levels of REDD1 as well as inflammatory cytokines, respectively. Cell apoptosis was observed with TUNEL staining. The expression of autophagy-related genes was detected by Western blot. Autophagy level was observed with GFP-LC3 staining. PM2.5 induced the expression of REDD1 in BEAS-2B cells. The inhibition by silencing REDD1 ameliorated the viability damage, blocked the inflammatory response and reduced the number of apoptotic BEAS-2B cells all induced by PM2.5. It was also found that PM2.5 induced autophagy in BEAS-2B cells, which was reversed by interference with REDD1. Furthermore, interference with REDD1 alleviated PM2.5-induced cell damage, inflammatory response and apoptosis in BEAS-2B cells through inhibiting autophagy. REDD1/autophagy inhibition ameliorates PM2.5-induced viability damage, inflammation and apoptosis in BEAS-2B cells.

## Graphical Abstract

**Figure:**
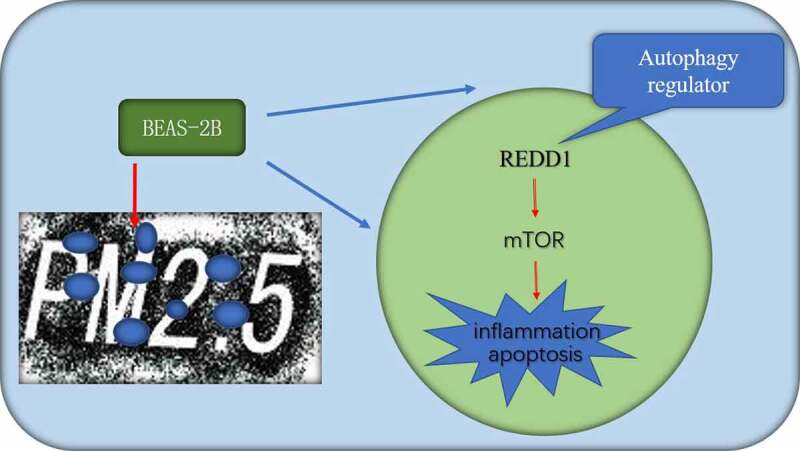

## Introduction

Lung cancer is the most commonly diagnosed cancer among all cancers in the world, and it accounts for the largest number of cancer deaths each year in both genders [1, 2]. Many would take it for granted that one will free from lung cancer threats so long as they are nonsmokers, and the pathogenesis of lung cancer, however, is much more than smoking [3]. Fine particulate matter refers to particulate matter with aerodynamic equivalent diameter of 2.5 microns or less in ambient air, which is also known as PM2.5. Substantial studies have proven the involvement of long-term PM2.5 inhalation in the occurrence of lung cancer [4]. It has also been corroborated that exposure to PM2.5 aggravates inflammatory response in the airway, leading to asthma, chronic obstructive pulmonary disease (COPD) and lung injuries, as well as long-term lag influences on the incidence of lung cancer [5, 6]. Research on the association between PM2.5 and children’s pulmonary function provides evidence that long-term exposure to outdoor or indoor PM2.5 induces and worsens asthmatic symptoms in pediatric asthma and has major adverse impacts on the overall lung function of children [7, 8]. Generally speaking, PM2.5 is a real threat to the health of people of all ages, including infants, children and adolescents. Various environmental problems that cause PM2.5 production need to be addressed, while the medical community is dedicated to exploring new ways to diagnose and treat the respiratory diseases attributed to air pollution. REDD1, regulated in development and DNA damage responses 1, is a key regulator protein that induces autophagy [9]. According to the current research, REDD1 participates in the process of COPD aggravation and is overly expressed in A549 alveolar type II epithelial cells under stimulation with hypoxia and dexamethasone [10, 11]. However, its role in fine particulate matter-induced lung injury has not been studied. Moreover, autophagy has been found to be significantly enhanced in PM2.5-induced lung injury [12]. This study is expected to serve as an inspiration for the development of the therapeutic strategies for PM2.5-induced lung injury as well as other diseases of the respiratory system. Based on the above facts, we hypothesized that REDD1 played a role in PM2.5-induced lung injury, which could be related to the regulation for mTOR axis. Therefore, an *in vitro* research was performed to explore the role of REDD1 and its regulatory mechanism in BEAS-2B cell injury induced by airborne particulate matter.

## Materials and methods

### Cell culture and treatment

We consulted the experiments of previous studies and selected BEAS-2B cells and PM2.5 in 100 μg/ml for the establishment of the cellular model of PM2.5-induced lung injury [13]. BEAS-2B cells were sourced from American Type Culture Collection and were grown in BEGM™ Bronchial Epithelial Cell Growth Medium (Lonza Group Ltd., Basel, Switzerland) in 95% air and 5% CO_2_ at 37°C.

### CCK-8

PM2.5-treated BEAS-2B cell viability was detected by Cell Counting Kit-8 (Beyotime, Nanjing, China). Cell suspension was seeded into a 96-well plate at a density of 100 μL per well (1 × 10^5^ cells) and was incubated in a humidified incubator in 5% CO_2_ at 37°C. After the addition of 10 μL of CCK-8 solution, the plate was incubated for 2 h. The optical density (OD) was measured by a microplate reader at 450 nm wavelength.

### RT-qPCR

The mRNA expressions of REDD1 and inflammatory cytokines were detected by RT-qPCR. Adherent cells were digested by trypsin, collected into a 3.5 cm culture dish and washed with PBS (Beyotime, Nanjing, China) to remove the medium. 1 mL TRIzol reagent (Invitrogen, Carlsbad, CA, USA) was added to the dish for the lysis of the cells. After total RNA was obtained, its density and purity were determined by an ultraviolet spectrophotometer. Reverse transcription was performed with Premix Taq^TM^ (TaKaRa, Tokyo, Japan) in accordance with the manufacturer’s protocol to synthesize cDNA which was then used for PCR amplification. The primer sequences are as follows: REDD1, forward, 5'-TGAGGATGAACACTTGTGTGC-3', reverse, 5'- CCAACTGGCTAGGCATCAGC-3'. TNF-α, 5'- −3' TNF-α, forward, 5'-AGGCAATAGGTTTTGAGGGCCAT-3', reverse, 5'-TCCTCCCTGCTCCGATTCCG-3'. IL-1β, forward, 5'-ATAAGCCCACTCTACACCT-3', reverse, 5'-ATTGGCCCTGAAAGGAGAGA-3'. IL-6, forward, 5'-ACTCACCTCTTCAGAACGAATTG-3', reverse, 5'-CCATCTTTGGAAGGTTCAGGTTG-3'. GAPDH, GAPDH forward, 5'-TGACGTGCCGCCTGGAGAAC-3', reverse, 5'-CCGGCATCGAAGGTGGAAGAG-3'. Real-Time PCR was performed on the StepOnePlus Real-Time PCR System. The relative levels were calculated with 2-△△Ct method.

### siRNA transfection

BEAS-2B cells were seeded into 6-well plate (5 × 10^5^ cells/well) and cultured for 24 h. Then, cells were transfected with Si-REDD1 (50pmol) or Si-NC using Lipofectamine 3000 (ThermoFisher Scientific Inc). After 4 h, cells were exposed to PM2.5 (50 μg/ml) for 24 h for further experiment. Si-REDD1 or Si-NC was constructed and purchased from GenePharma (Shanghai, China). The sequences of Si-REDD1 are as following: Sense: 5'-UGGUAAGCCAGGUGGGCAA-3' and Antisense: 5'-UUGCCCACCUGGCUUACCA-3'.

### Western blot

The expression of REDD1, inflammatory cytokines, apoptosis-related proteins and autophagy proteins were determined by Western blot analysis. For total protein extraction, 3 ml pre-cooled PBS was added to wash the cells three times to rinse off the culture medium. 400 μL of RIPA lysis buffer containing PMSF (Sigma-Aldrich, St. Louis, MO, USA) was added to each flask to lysate the cells on ice for 30 min. After centrifugation at 2024.8 × g for 5 min at 4°C, the supernatant was stored in a 0.5 ml tube at −20°C. The protein concentration was examined by Enhanced BCA Protein Assay Kit (Beyotime, Nanjing, China). Subsequently, SDS-PAGE electrophoresis was performed before transference to a PVDF membrane (Corning Incorporated, Corning, NY, USA). Next, TBST-diluted primary antibody (REDD1, ab106356, 1:1000; p65, ab32536, 1:1000; COX2, ab179800, 1:1000; Bcl-2, ab32124, 1:1000; Bax, ab32503, 1:2000; ab32042, 1:500; Cleaved Caspase3, ab32042, 1:500; Cleaved Caspase9, ab2324; 1:1000; Cleaved PARP, ab32064, 1:1000; Beclin-1, ab210498, 1:1000; ATG7, ab52472, 1:100000; LC3, ab192890, 1:2000; p62, ab109012, 1:20000; p-mTOR, ab109268, 1:1000; mTOR, ab134903, 1:10000; GAPDH, cat. no. ab8245, 1:5000, Abcam, England) was incubated with the membrane for 2 h before washing with TBST (Thermo Fisher Scientific, Waltham, MA, USA) twice on a shaker for 10 min each time, followed by QuickBlock™ Blocking Buffer (Beyotime, Nanjing, China) for 10 min. The membrane was then incubated with diluted secondary antibody (Goat anti-Rabbit IgG, ab216777, 1:10000, Abcam, England) for 2 h at room temperature and washed in the same manner. Chemiluminescence was conducted in the end. The gray value of protein bands was analyzed using Image J 1.46 r software (National Institutes of Health).

### TUNEL staining

PM2.5-induced BEAS-2B cell apoptosis was detected using TUNEL Apoptosis Assay Kit (Beyotime, Nanjing, China) under the operation guidelines provided by the manufacturer. The adherent cells were washed once with PBS and fixed with 4% paraformaldehyde (Macklin, Shanghai, China) for 30 min. PBS containing 0.3% Triton X-100 was added to incubate the cells for 20 min at room temperature. TUNEL solution was prepared with reagents in the kit. 50 μl of TUNEL solution was added to each sample for incubation in the dark for 60 min at 37°C. The apoptotic cells were observed under a fluorescence microscope (Roche, Switzerland) after anti-fluorescence quenching sealing solution was used to seal the sections.

### GFP-LC3 staining

The BEAS-2B cells were collected and fixed with 100% methanol (5 min). Next, cells were subjected to the permeabilization of 0.1% Triton X-100 for 5 min and blocked with 10% normal goat serum 0.1% PBS-Tween for 1 h. Then, cells were performed incubation with primary antibody (LC3, 1:50, ab192890) overnight at 4°C, followed by an incubation at room temperature for 1 h with a goat secondary antibody to Rabbit IgG (ab150081). DAPI was used to stain nuclear DNA.

### Statistical analysis

All experiments were repeated at least in triplicate. Results are presented as mean ± S.D. one-way ANOVA was applied for the statistical comparisons among different treatment groups, followed by Tukey’s test for comparisons between the two groups. For all data, P < 0.05 was defined as statistical significance.

## Results

### PM2.5 induces increased expression of REDD1 in BEAS-2B

**Figure 1.**
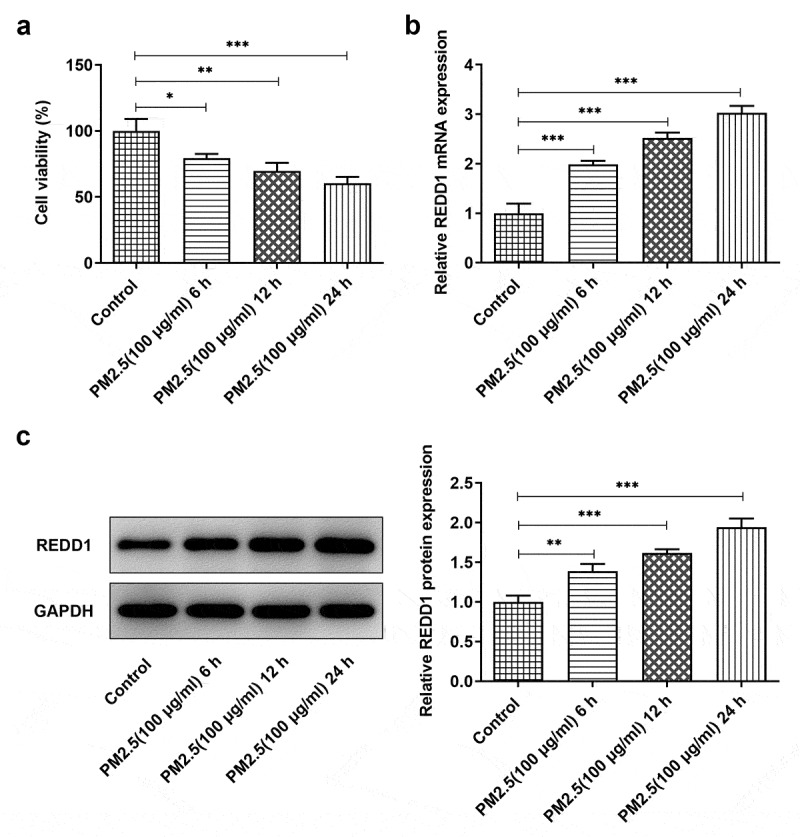
PM2.5 induces increased expression of REDD1 in BEAS-2B

To define the correlation between PM2.5 exposure and the expression of REDD1, we treated BEAS-2B cells with 100 μg/ml PM2.5 for 6, 12, or 24 h, respectively. Detection with CCK-8 showed declined BEAS-2B cell viability with the increase in PM2.5 treatment time (Figure 1(a)). REDD1 expression detected by RT-qPCR andWestern blot demonstrated the opposite trend where REDD1 expression increased as the time went by (Figure 1(b-c)). PM2.5 (100 μg/ml) 24 h group exhibited the highest expression of REDD1 and was thus selected for the subsequent experiments. These results indicate a positive correlation between REDD1 expression and PM2.5 exposure.

(A) The viability of BEAS-2B cells after PM2.5 inducement for 6, 12, and 24 h, detected by CCK-8. (B-C) REDD1 expression in BEAS-2B cells after PM2.5 inducement for 6, 12, and 24 h, detected by RT-qPCR and Western blot. *p < 0.05, **p < 0.01, ***p < 0.001.

### REDD1 inhibition ameliorates PM2.5-induced viability damage and inflammatory response in BEAS-2B cells

**Figure 2.**
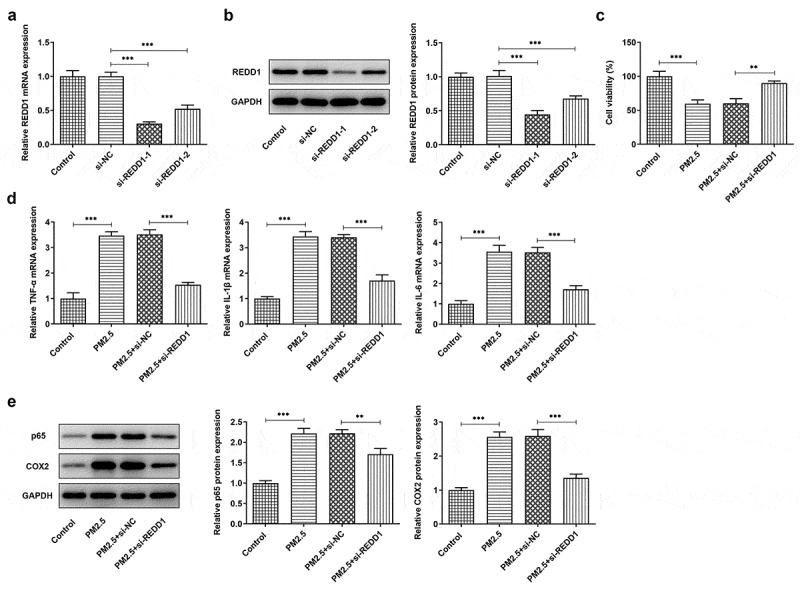
REDD1 inhibition ameliorates PM2.5-induced viability damage and inflammatory response in BEAS-2B cells

To preliminarily explore the role of REDD1 in PM2.5-induced lung injury, we constructed the small interfering RNA plasmids of REDD1 (si-REDD1-1 and si-REDD1-2) for transfection into PM2.5-treated BEAS-2B cells, followed by RT-qPCR and Western blot to determine REDD1 expression. The result showed decreased expression of REDD1 in transfected cells where si-REDD1-1 exhibited the lower REDD1 expression than si-REDD1-2 (Figure 2(a-b)). Therefore, si-REDD1-1 was selected for the subsequent experiments. si-REDD1-1 or si-NC was used to transfect BEAS-2B cells for 4 h, which then were exposed to PM2.5 for 24 h. CCK-8 was used to detect cell viability, and it was found that while BEAS-2B cell viability was greatly dampened by PM2.5 exposure by contrast with the control, it was improved again by transfection with si-REDD1 (Figure 2(c)). The expressions of inflammatory cytokines such as TNF-α, IL-1β and IL-6 in BEAS-2B cells detected by RT-qPCR showed significant increase after PM2.5 treatment, which were attenuated to a great extent by transfection with siREDD1 (Figure 2(d)). Western blot further verified the result of RT-qPCR, which also detected increased expression of NF-kappaB p65 and COX-2 – two inflammation-related proteins in PM2.5-treated BEAS-2B cells and decreased expression of those by si-REDD1 transfection (Figure 2(e)). Collectively, these results demonstrate the inhibitory role of REDD1 interference in PM2.5-induced BEAS-2B cell viability damage and inflammatory response.

(A-B) REDD1 expression in BEAS-2B cells transfected with control, si-NC, si-REDD1-1 or si-REDD1-2, detected by RT-qPCR and Western blot. (C) The viability of PM2.5-treated BEAS-2B cells before and after transfection with si-REDD1, detected by CCK-8. (D) The expressions of pro-inflammatory cytokines TNF-α, IL-1β and IL-6 in PM2.5-treated BEAS-2B cells before and after transfection with si-REDD1, detected by RT-qPCR. (E) The expressions of proteins NF-kappaB p65 and COX-2 in PM2.5-treated BEAS-2B cells before and after transfection with si-REDD1, detected by Western blot. **p < 0.01, ***p < 0.001.

### REDD1 inhibition attenuates PM2.5-induced BEAS-2B cell apoptosis

**Figure 3.**
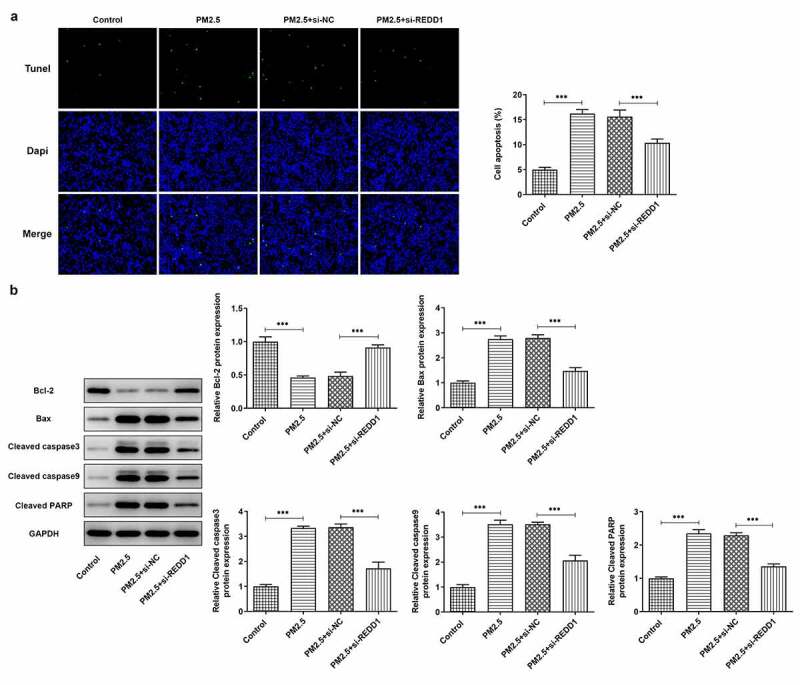
REDD1 inhibition attenuates PM2.5-induced BEAS-2B cell apoptosis

To further explore the role of REDD1 in PM2.5-induced lung injury, we made a comparison between the apoptosis level of PM2.5-induced BEAS-2B cells before and after transfection with si-REDD1. TUNEL staining observed much higher apoptosis level of PM2.5-induced BEAS-2B cells than that in the control group and that transfection with si-REDD1 significantly suppressed the increased apoptosis level induced by PM2.5 (Figure 3(a)). Western blot analysis determined increased gene expressions of pro-apoptotic proteins such as Bax, cleaved caspase3, cleaved caspase9 and cleaved PARP and decreased expression of anti-apoptotic protein Bcl2 in PM2.5-treated BEAS-2B cells, which were all reversed conspicuously by transfection with si-REDD1 (Figure 3(b)). These results suggest that REDD1 interference has an inhibitory effect on the increased BEAS-2B cell apoptosis induced by PM2.5 exposure.

(A) The apoptosis of PM2.5-treated BEAS-2B cells before and after transfection with si-REDD1, observed with TUNEL staining. (B) The expressions of apoptotic proteins Bax, cleaved caspase3, cleaved caspase9, cleaved PARP and Bcl2 in PM2.5-treated BEAS-2B cells before and after transfection with si-REDD1, detected by Western blot. ***p < 0.001.

### REDD1 interference inhibits PM2.5-induced autophagy expression in BEAS-2B cells

**Figure 4.**
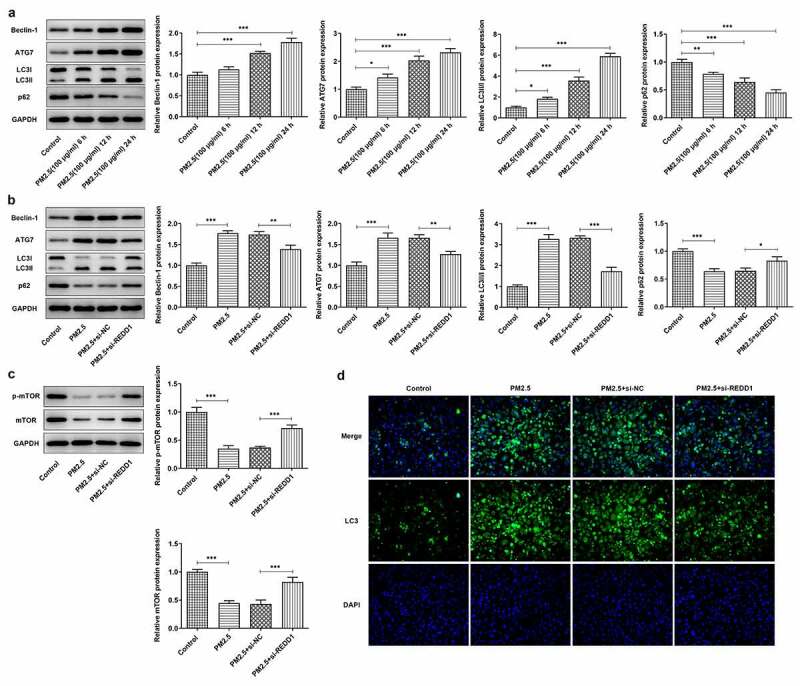
REDD1 interference inhibits PM2.5-induced autophagy expression in BEAS-2B cells

For the purpose of finding out the relationship between PM2.5 and autophagy expression in PM2.5-induced lung injury and whether REDD1 impacts on it, the autophagy expression was examined in PM2.5-treated BEAS-2B cells. The expression of autophagy-specific proteins was detected by Western blot, where the expression of beclin-1, ATG7 and LC3 was elevated and that of P62 and LC3I declined with the increase in PM2.5 treatment time (Figure 4(a)). It was reported that PM2.5 induced increased autophagy levels and upregulated the levels of autophagy-related proteins [14, 15]. After transfection with si-REDD1 into BEAS-2B cells, it was found that the expression of beclin-1, ATG7 and LC3II was reduced and that of P62 and LC3I was increased by comparison with their expression in those cells without REDD1 interference (Figure 4(b)). It was also found that the expression of autophagy regulator p-mTOR/mTOR in BEAS-2B cells was decreased by PM2.5 exposure, which was reversed to a great extent by transfection with si-REDD1 (Figure 4(c)). Furthermore, GFP-LC3 staining observed that exposure to PM2.5 increased the autophagy level of BEAS-2B cells, which was significantly decreased after REDD1 interference (Figure 4(d)). Taken together, these results indicate that interference with REDD1 expression can suppress the increased autophagy level in BEAS-2B cells induced by exposure to PM2.5.

(A) The expressions of autophagy-related proteins in BEAS-2B cells after PM2.5 inducement for 6, 12, and 24 h, detected by Western blot. (B) The expressions of autophagy-related proteins in PM2.5-treated BEAS-2B cells before and after transfection with si-REDD1, detected by Western blot. (C) The expression of p-mTOR and mTOR in PM2.5-treated BEAS-2B cells before and after transfection with si-REDD1, detected by Western blot. (D) The autophagy level of PM2.5-treated BEAS-2B cells before and after transfection with si-REDD1, observed with GFP-LC3 staining. *p < 0.05, **p < 0.01, ***p < 0.001.

### REDD1 interference ameliorates PM2.5-induced viability damage and inflammatory injury in BEAS-2B cells via inhibiting autophagy

**Figure 5.**
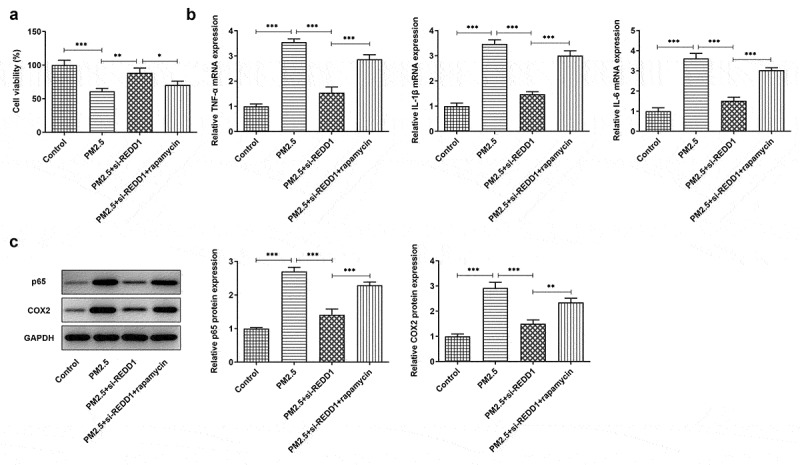
REDD1 interference ameliorates PM2.5-induced viability damage and inflammatory injury in BEAS-2B cells via inhibiting autophagy

To identify the mechanism behind the effect of REDD1 inhibition on PM2.5-induced viability damage and inflammatory response in BEAS-2B cells, 10 nM rapamycin (Sigma-Aldrich, St. Louis, MO, USA) was chosen for inhibition of mTOR and promotion of autophagy. The viability of BEAS-2B cells detected by CCK-8 was found to be largely weakened by PM2.5 exposure, comparatively improved by REDD1 inhibition and decreased again by rapamycin treatment (Figure 5(a)). According to the result of RT-qPCR, increased mRNA expression of pro-inflammatory cytokines such as TNF-α, IL-1β and IL-6 was suppressed by si-REDD1 transfection in PM2.5-treated BEAS-2B cells, which was reversed after rapamycin treatment (Figure 5(b)). Similarly, increased gene expression of inflammation-related NF-kappaB p65 and COX-2 was also downregulated by REDD1 inhibition in PM2.5-treated BEAS-2B cells, which was reversed by rapamycin treatment (Figure 5(c)). These results imply that interference with REDD1 expression may inhibit autophagy and thereby ameliorates PM2.5-induced BEAS-2B cell viability damage and inflammatory injury.

(A) The viability of BEAS-2B cells treated with control, PM2.5, PM2.5+ si-REDD1 or PM2.5+ si-REDD1+ rapamycin, detected by CCK-8. (B) The expressions of pro-inflammatory cytokines TNF-a, IL-1β and IL-6 in BEAS-2B cells treated with control, PM2.5, PM2.5+ si-REDD1 or PM2.5+ si-REDD1+ rapamycin, detected by RT-qPCR. (C) The expressions of NF-kappaB p65 and COX-2 in BEAS-2B cells treated with control, PM2.5, PM2.5+ si-REDD1 or PM2.5+ si-REDD1+ rapamycin, detected by Western blot. *p < 0.05, **p < 0.01, ***p < 0.001.

### REDD1 interference ameliorates PM2.5-induced BEAS-2B cell apoptosis via inhibiting autophagy

**Figure 6.**
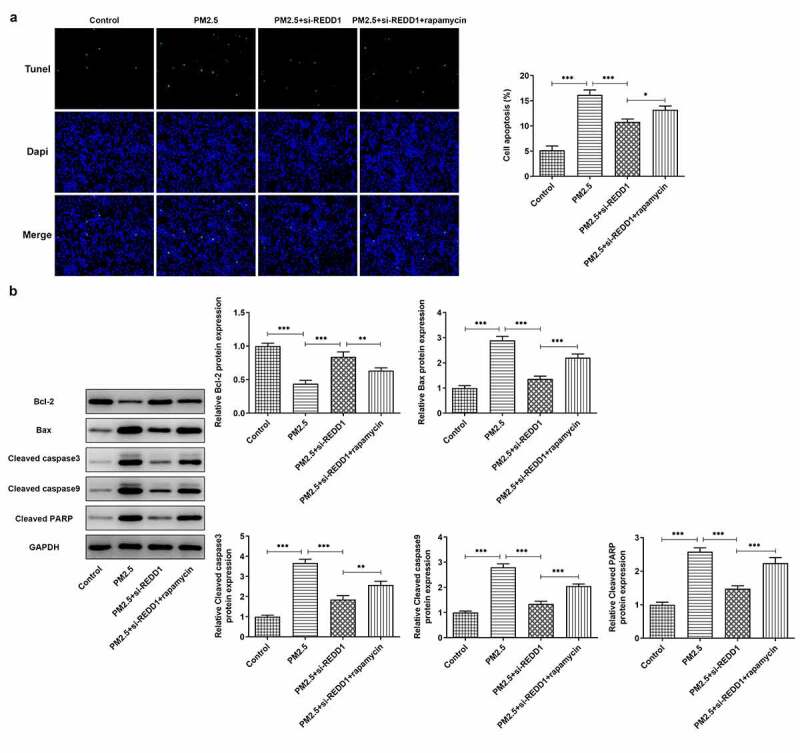
REDD1 interference ameliorates PM2.5-induced BEAS-2B cell apoptosis via inhibiting autophagy

We carried out experiments on how interaction between REDD1 and autophagy affects PM2.5-induced BEAS-2B cell apoptosis. It was observed through TUNEL staining that REDD1 inhibition alleviated PM2.5-induced BEAS-2B cell apoptosis, which was again aggravated by rapamycin treatment (Figure 6(a)). The expression of pro-apoptotic Bax, cleaved caspase3, cleaved caspase9 and cleaved PARP increased by PM2.5 exposure was inhibited by REDD1 interference, which was then reversed after treatment with rapamycin in BEAS-2B cells (Figure 6(b)). The expression of anti-apoptotic Bcl2, however, exhibited the opposite changes (Figure 6(b)). It suggests that REDD1 inhibition ameliorates PM2.5-induced BEAS-2B cell apoptosis, most likely through suppressing autophagy level.

(A) The apoptosis of BEAS-2B cells treated with control, PM2.5, PM2.5+ si-REDD1 or PM2.5+ si-REDD1+ rapamycin, observed with TUNEL staining. (B) The expressions of Bax, cleaved caspase3, cleaved caspase9, cleaved PARP and Bcl2 in BEAS-2B cells treated with control, PM2.5, PM2.5+ si-REDD1 or PM2.5+ si-REDD1+ rapamycin, detected by Western blot. *p < 0.05, **p < 0.01, ***p < 0.001.

## Discussion

With global population growth, urbanization, land desertification and the aggravation of industrial pollution, the average concentration of PM2.5 is constantly rising around the world, and the resulting environmental and health problems have aroused great concern [16]. It is supported by substantial research that long-term exposure to PM2.5 increases the risk of developing serious respiratory diseases, including cancer, among all ages [17]. Children, as may easily be imagined, are also sufferers from air pollution-induced respiratory diseases. For example, it is found that PM2.5 exposure augments respiratory inflammation response and oxidative stress, eventually causing asthmatic attack in children [18]. In China, domestic research has revealed increased relative risk of pediatric asthma hospital visits, as a short-term result of high concentration of ambient PM2.5 [19]. Hence, how to treat or alleviate lung injury and other respiratory lesions caused by PM2.5 has already become a popular direction for medical research in recent years [20–22].

The process of type II programmed cell death, namely autophagy, is closely implicated in the pathophysiology of non-small-cell lung cancer as well as the pathogenesis of many other pulmonary diseases, which contributes to protein degradation and cell apoptosis [23, 24]. Studies have corroborated elevated autophagy level in PM2.5-induced lung injury and pulmonary inflammatory responses [12, 25]. Increased autophagy level was also detected in our study in PM2.5-treated BEAS-2B cells, which is consistent with previous studies. REDD1 is an autophagy-related protein that regulates autophagy level in various types of diseases [26, 27]. A study reported the involvement of REDD1 in the aggravated progression of chronic obstructive pulmonary disease [10]. Additionally, REDD1 overexpression was observed in alveolar type II epithelial cells under stimulation of hypoxia and dexamethasone [11]. In our study, increased expression of REDD1 was detected in PM2.5-treated BEAS-2B cells. Furthermore, we also found that inhibiting REDD1 expression noticeably alleviates PM2.5-induced cell viability damage and inflammatory response in BEAS-2B cells by increasing cell viability, downregulating the expression of inflammatory cytokines and attenuating cell apoptosis.

Beclin-1, ATG7 and LC3 are autophagy-specific proteins, the accumulated expression of which will lead to autophagy ignition [28, 29]. In our study, the expression of these proteins showed significant promotion by PM2.5 exposure and great reduction by REDD1 interference. P62 is an autophagy receptor, which is normally produced and degraded constantly by selective autophagy [30]. Its expression was found by us to be decreased by PM2.5 exposure and increased after REDD1 inhibition. Therefore, our study demonstrated that PM2.5 induces accumulation of autophagy and that REDD1 inhibition can suppress autophagy level in BEAS-2B cells. Mammalian/mechanistic target of rapamycin (mTOR) is another regulator of autophagy [31–33]. It has already been proved that mTOR signaling activation can effectively inhibit lung fibroblast autophagy as well as autophagy in many other diseases [34, 35]. Rapamycin, an mTOR inhibitor, has been shown to promote autophagy in pulmonary arterial smooth muscle cells by inhibiting mTOR signaling [36]. Furthermore, interference with REDD1 has been shown to attenuate ischemic injury in neuron by inhibiting mTOR-regulated autophagy level with the use of rapamycin [37]. Based on these studies, we also selected rapamycin to inhibit mTOR to further validate the action mechanism of REDD1 interference. It was found that while the viability damage, the inflammatory response and the apoptosis level in PM2.5-treated BEAS-2B cells were all incredibly ameliorated by REDD1 interference, rapamycin treatment reversed such effect to a great extent. It suggests that REDD1 inhibition suppresses the level of autophagy to alleviate the viability damage, inflammatory response and apoptosis in PM2.5-treated BEAS-2B cells.

## Conclusion

To sum up, data in our study support that inhibiting REDD1/autophagy alleviates PM2.5-induced inflammation and apoptosis in BEAS-2B cells. The present study enlightens a new direction for the research on treatment for PM2.5-induced lung injury. As for practical application, more studies on the action mechanism of REDD1/autophagy are warranted in the future.

## Data Availability

The datasets used and analyzed are available from the corresponding author and first author on reasonable request.

## Highlights

1. REDD1 mediated the enhanced autophagy levels of BEAS-2B cells induced by PM2.5.
2. REDD1 regulated mTOR axis in BEAS-2B cells exposed to PM2.5.
3. REDD1 silencing suppressed inflammation and apoptosis in BEAS-2B cells with PM2.5 induction through mTOR axis.
